# Remote Heart Rhythm Monitoring by Photoplethysmography-Based Smartphone Technology After Cardiac Surgery: Prospective Observational Study

**DOI:** 10.2196/26519

**Published:** 2021-04-15

**Authors:** Marie Lamberigts, Lucas Van Hoof, Tine Proesmans, Pieter Vandervoort, Lars Grieten, Peter Haemers, Filip Rega

**Affiliations:** 1 Department of Cardiac Surgery University Hospitals Leuven Leuven Belgium; 2 Qompium NV Hasselt Belgium; 3 Department of Cardiology Ziekenhuis Oost-Limburg Genk Belgium; 4 Department of Cardiology University Hospitals Leuven Leuven Belgium

**Keywords:** cardiac surgery, postoperative follow-up, cardiac rehabilitation, postoperative arrhythmias, atrial fibrillation, photoplethysmography, home-monitoring

## Abstract

**Background:**

Atrial fibrillation (AF) is the most common arrhythmia after cardiac surgery, yet the precise incidence and significance of arrhythmias after discharge home need to be better defined. Photoplethysmography (PPG)-based smartphone apps are promising tools to enable early detection and follow-up of arrhythmias.

**Objective:**

By using a PPG-based smartphone app, we aimed to gain more insight into the prevalence of AF and other rhythm-related complications upon discharge home after cardiac surgery and evaluate the implementation of this app into routine clinical care.

**Methods:**

In this prospective, single-center trial, patients recovering from cardiac surgery were asked to register their heart rhythm 3 times daily using a Food and Drug Administration–approved PPG-based app, for either 30 or 60 days after discharge home. Patients with permanent AF or a permanent pacemaker were excluded.

**Results:**

We included 24 patients (mean age 60.2 years, SD 12 years; 15/23, 65% male) who underwent coronary artery bypass grafting and/or valve surgery. During hospitalization, 39% (9/23) experienced postoperative AF. After discharge, the PPG app reported AF or atrial flutter in 5 patients. While the app notified flutter in 1 patient, this was a false positive, as electrocardiogram revealed a 2nd-degree, 2:1 atrioventricular block necessitating a permanent pacemaker. AF was confirmed in 4 patients (4/23, 17%) and interestingly, was associated with an underlying postoperative complication in 2 participants (pneumonia n=1, pericardial tamponade n=1). A significant increase in the proportion of measurements indicating sinus rhythm was observed when comparing the first to the second month of follow-up (*P*<.001). In the second month of follow-up, compliance was significantly lower with 2.2 (SD 0.7) measurements per day versus 3.0 (SD 0.8) measurements per day in the first month (*P*=.002). The majority of participants (17/23, 74%), as well as the surveyed primary care physicians, experienced positive value by using the app as they felt more involved in the postoperative rehabilitation.

**Conclusions:**

Implementation of smartphone-based PPG technology enables detection of AF and other rhythm-related complications after cardiac surgery. An association between AF detection and an underlying complication was found in 2 patients. Therefore, smartphone-based PPG technology may supplement rehabilitation after cardiac surgery by acting as a sentinel for underlying complications, rhythm-related or otherwise.

## Introduction

Postoperative atrial fibrillation (POAF) is one of the most important complications after cardiac surgery, occurring in 10%-65% of patients, depending on their risk profile and surgery type [1-3]. The burden of this complication on health care and the economy is significant, as it leads to elevated stroke risk, higher postoperative mortality, longer hospitalization, and an increase in medical costs [3-5]. AF and other arrhythmias typically occur early after surgery, with the incidence of AF peaking on the second to third postoperative day, correlating with the inflammatory peak [2,6,7]. In studies with extensive follow-up using noninvasive telemetry or invasive event recorders, late-onset POAF occurs in approximately 9% of patients [8-13]. Recurrent POAF is more common, with an estimated recurrence rate of 30% in the first months postoperatively [14,15]. The precise incidence of late-onset or recurrent AF remains uncertain due to discrepancies between previous studies.

The basic principle of photoplethysmography (PPG) technology is the detection by an optical sensor of variations in light intensity, as reflected by or transmitted through tissue. The tissue perfusion with every heartbeat can be registered, and by extrapolating the RR-interval, the underlying heart rhythm can be determined. FibriCheck (Qompium NV, Hasselt, Belgium) is an example of a Conformité Européenne– and Food and Drug administration–approved smartphone app that employs the phone’s flashlight and camera, thereby offering a low-budget and widely accessible platform for early diagnosis and close follow-up of AF [16,17]. Previous studies have extensively confirmed its accuracy in detecting AF and the feasibility of use in a primary care setting or as a large-scale screening tool [17-19]. While there are currently no reports on the systematic use of a PPG-based smartphone app after cardiac surgery, such an approach is promising to detect late-onset or recurrent POAF that may otherwise be missed in routine clinical follow-up. On the other hand, the importance and therapeutic implications of these sometimes short and asymptomatic episodes of AF are uncertain. Therefore, the potential added value of PPG technology in postoperative rehabilitation must be evaluated.

The objective of this study was to gain insight into the prevalence of arrhythmias upon discharge home after cardiac surgery by using a PPG-based app. Furthermore, we aimed to evaluate the added value and obstacles to implementation of this app into routine clinical care.

## Methods

### Study Design

This study was constructed as an observational, monocentric cohort study. An a priori sample size calculation was not performed for this proof-of-concept trial. This study was approved by the Ethics Committee Research of University Hospitals (UZ)/Katholieke Universiteit Leuven (S63159) and was conducted in accordance with the Declaration of Helsinki.

At discharge, participants were educated on the use of the app by a member of the research team before starting a follow-up period of 60 days in which they used FibriCheck to assess their heart rhythm. The home monitoring consisted of 3 measurements per day, with each measurement being 1 minute long. If compliance decreased, the participant was sent a reminder. At the end of the study period, participants were contacted and asked to complete a short questionnaire on their personal experience of using the smartphone app as a follow-up tool.

### FibriCheck App and Evaluation of PPG Registrations

The specific PPG-based smartphone app under evaluation was FibriCheck, which is capable of distinguishing multiple different arrhythmias. Measurements are performed by placing a finger over the camera of a smartphone for 1 minute. During this period, the smartphone’s optical sensor detects variations in tissue perfusion related to the heart rhythm, enabling extrapolation of the RR-interval. An example of a measurement is provided in Multimedia Appendix 1. Each measurement is then uploaded to a server and immediately analyzed by a dedicated algorithm, classifying the measurement into 1 of 4 categories (normal, warning, urgent, insufficient quality). Warning measurements include non-AF arrhythmias, and urgent measurements include possible AF arrhythmias. Measurements indicating normal sinus rhythm are validated immediately by the algorithm, whereas measurements with possible irregularities are reviewed by highly trained personnel within, at most, 48 hours. As specified in the instructions for use, every arrhythmia detected by FibriCheck should be confirmed by an electrocardiogram (ECG).

**Figure 1:**
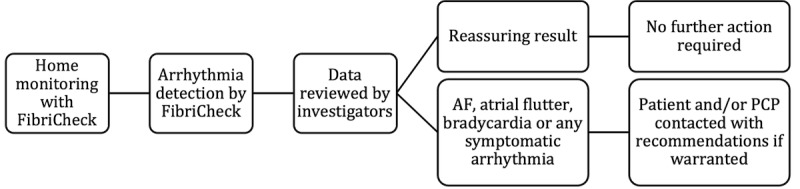
Schematic overview of the protocol for management of detected arrhythmias during the follow-up phase. AF: atrial fibrillation; PCP: primary care physician.

As per standard protocol for FibriCheck, all PPG measurements were immediately available to the research team through the online dashboard of the app. To prevent delays in diagnosis, the investigators checked this dashboard daily for abnormal measurements. If an unconfirmed measurement was suspected by the research team to indicate AF, the study participant was contacted, and the research team suggested that they visit their primary care physician (PCP). Furthermore, patients were instructed at study inclusion to seek emergent medical care if they felt unwell and not rely on the app to guide them. An overview of the protocol in case of arrhythmia detection is shown in Figure 1.

### Outcome Measures

The primary outcome was the detection of postoperative arrhythmias after hospital discharge, mainly focusing on late-onset or recurrent postoperative AF. Secondary outcomes included the detection of other rhythm-related events, the impact and added value of FibriCheck on routine postoperative care, and the experience of patients and PCPs.

### Participant Recruitment and Eligibility Criteria

Patients were recruited during their recovery period after cardiac surgery at UZ Leuven. Inclusion criteria consisted of (1) a minimum age of 18 years, (2) possession of a smartphone, (3) underwent cardiac surgery at UZ Leuven, and (4) written informed consent. Exclusion criteria were (1) permanent AF; (2) pacemaker-dependent heart rhythm or ventricular assist device; (3) significant cognitive impairment (eg, dementia); (4) poor finger perfusion (eg, intensive callus formation, perniosis); (5) tremor, Parkinson’s disease, or other disabilities resulting in the inability to perform measurements; (6) nonnative Dutch speakers; and (7) long and complicated postoperative hospitalization, for example after endocarditis surgery.

### Data Collection and Analysis

Data were collected and processed anonymously, in accordance with regulations on data protection, ethics, and written informed consent. Patient-related information was retrieved from the electronic medical records system used at UZ Leuven, while data on the PPG registrations were exported from the FibriCheck app interface.

Continuous variables are presented as mean (SD), and categorical variables are presented as numbers and percentages. The Shapiro-Wilk test was used to test normality. The Student *t* test, Wilcoxon rank sum test, and Fisher exact test were used to determine significance when comparing variables between subgroups. The 2-proportion z test was used to determine the significance of proportions between subgroups. Statistical significance was set at *P*<.05, and data were analyzed using R Studio version 1.1.447 (RStudio Inc, Boston, MA) or Microsoft Excel for Mac version 16.36 (Microsoft Corporation, Redmond, WA).

## Results

### Study Population

Between January 29, 2020 and March 12, 2020, patients recovering from cardiac surgery at UZ Leuven were screened for participation. In this period, 24 patients were initially enrolled in the study. A total of 23 participants completed the study period, as 1 participant received a permanent pacemaker for late-onset, second-degree atrioventricular (AV) block with 2:1 conduction. All remaining 23 participants were home monitored for 30 days, and 17 were followed for an additional 30 days. The mean age was 60.2 (SD 12) years, and the majority of the study population was male (15/23, 65%). Table 1 provides an overview of demographic and other variables of the study population.

**Table 1:** Overview of (demographic) variables of the study population (n=23).

Variables		Values
Age (years), mean (SD)		60.2 (12)
Gender (male), n (%)		15 (65)
BMI (kg/m^2^), mean (SD)		26.5 (5)
Diabetes, n (%)		2 (9)
Hypertension, n (%)		9 (39)
Hypercholesterolemia, n (%)		7 (30)
BMI >30 kg/m^2^, n (%)		3 (13)
CHA_2_DS_2_-VASc^a^ score, mean (SD)		2 (1)
HAS-BLED^b^ score, mean (SD)		1 (1)
EuroScore II, mean (SD)		1.4 (1)
LVEF^c^, mean (SD)		58.2 (6)
LVDD^d^ (mm), mean (SD)		47.9 (8)
LAVI^e^ (mL/m^2^), mean (SD)		30.9 (8)
Length of ICU^f^ stay (days)^g^, mean (SD)		1.5 (2)
Total length of stay (days)^g^, mean (SD)		6.9 (3)
**Surgery type, n (%)**		
	AVR^h^/root/arch replacement	8 (35)
	CABG^i^	7 (30)
	Mitral valve surgery	5 (22)
	ASD^j^ closure	1 (4)
	CABG + AVR	1 (4)
	MVP^k^ + root replacement	1 (4)

^a^CHA_2_DS_2_-VASc: congestive heart failure, hypertension, age (>75 years), diabetes, stroke/transient ischemic attack, vascular disease, previous myocardial infarction, age (65-75 years), and sex category.

^b^HAS-BLED: hypertension, abnormal renal and liver function, stroke, bleeding, liable INR, elderly, drugs or alcohol.

^c^LVEF: left ventricle ejection fraction.

^d^LVDD: left ventricle diastolic diameter.

^e^LAVI: left atrial volume index.

^f^ICU: intensive care unit.

^g^Uz025 length of ICU stay is removed as an outlier; cut off boundaries were set at >20 days.

^h^AVR: aortic valve repair.

^i^CABG: coronary artery bypass grafting.

^j^ASD: atrium septum defect.

^k^MVP: mitral valve plasty.

During hospitalization, POAF occurred in 9 of the 23 (39%) patients, 4 of whom underwent isolated coronary artery bypass grafting surgery. The remaining 5 participants who experienced in-hospital POAF underwent various procedures. Other arrhythmias detected by telemetry during the postoperative hospitalization included several episodes of supraventricular tachycardia, sinus bradycardia, and frequent ventricular extrasystoles and one ventricular tachycardia episode.

A total of 42 patients were excluded prior to participation, and these patients were significantly older than the study population (average age of 71.7 vs 60.2 years, *P*=.002). Reasons for exclusion were lack of smartphone possession (n=13), patients that were transferred to other hospitals (n=9), permanent pacemaker (n=7), refusal (n=6), permanent AF (n=4), and nonnative Dutch-speaking patients (n=3).

### Overview of All FibriCheck Measurements

The total number of measurements performed by the 23 participants was 3271, with 2808 (2808/3271, 85.9%) being normal, 288 (288/3271, 8.8%) measurements labelled as a warning, and 27 (27/3271, 0.8%) prompted urgent action. The remaining 148 (148/3271, 4.5%) measurements were of insufficient quality. A majority of the warning measurements (159/288, 55.2%) occurred in one patient experiencing very frequent extrasystoles, and 67% (18/27) of all urgent measurements originated from one other patient. Concerning the diagnosis assigned to each measurement, 2792 (2792/3271, 85.4%) were sinus rhythm, 295 (295/3271, 9.0%) measurements were premature ventricular contractions, and bradyarrhythmias and tachyarrhythmias accounted for 5 (5/3271, 0.2%) and 2 (2/3271, 0.1%) of the measurements, respectively. Of all measurements, 27 (27/3271, 0.8%) were diagnosed as atrial flutter or fibrillation.

Most measurements (2929/3271, 89.5%) with the “normal” or “insufficient quality” label were automatically diagnosed as sinus rhythm or “insufficient quality” by the algorithm without further review by FibriCheck staff. The remaining 342 (342/3271, 10.5%) measurements, mostly warning and urgent measurements, were manually reviewed by FibriCheck staff. The mean timespan between measurement performance and review was 16.6 (SD 11.8) hours, and the maximum duration seen was 47.0 hours.

### Detection of Atrial Fibrillation

AF was confirmed in 4 participants (4/23, 17%) and interestingly, was associated with underlying complications in 2 participants. One 70-year-old male participant who experienced recurrent POAF 7 days after discharge simultaneously presented with a late-occurring pericardial tamponade. After surgical drainage, AF was not reported any more by the app. The second patient with an underlying complication was a 48-year-old man in whom FibriCheck detected late-onset AF on days 4 and 5 after discharge. After subsequent readmission, a diagnosis of pneumonia was made. He received intravenous antibiotics and underwent an electrical cardioversion.

### Detection of a Second-Degree AV Block

During follow-up, FibriCheck indicated bradycardia accompanied by frequent premature ventricular contractions in one participant during the first 9 days after discharge. This participant was a 56-year-old woman with Barlow’s disease who underwent minimally invasive mitral valve repair. Starting on the 10th day after discharge (postoperative day 15), the app indicated atrial flutter. This was found to be a false positive as an ECG revealed a 2nd-degree 2:1 AV block requiring a permanent pacemaker, excluding her for the remainder of the trial.

### First Versus Second Month of Follow-Up

**Figure 2:**
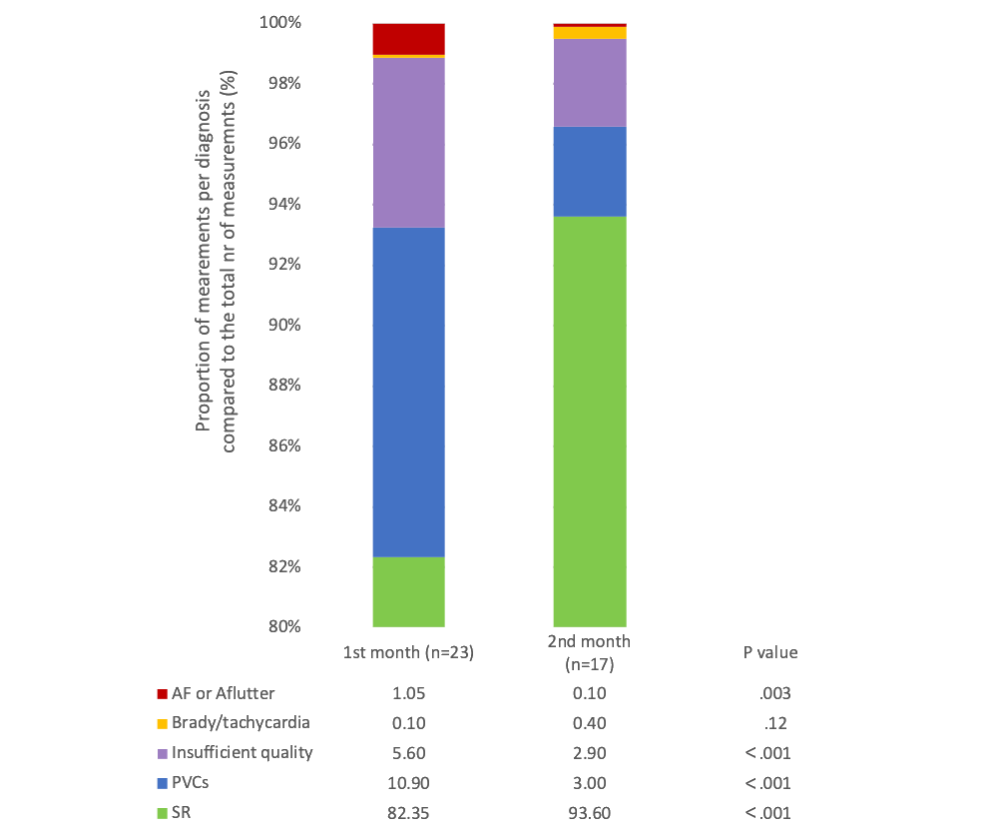
Overview of FibriCheck results comparing the proportion of each rhythm diagnosis between the first and second month of follow-up, with significance determined using 2-proportion z tests. AF: atrial fibrillation; PVC: premature ventricular contraction, SR: sinus rhythm.

All 23 participants were followed for 30 days, and 17 participants were followed for an additional 30 days. Table 2 depicts the differences between the first and second months of follow-up. Significantly fewer measurements were performed in total, and less were of insufficient quality. We observed a significant increase in the proportion of measurements diagnosed as sinus rhythm as well as a decrease in the prevalence of other arrhythmias (Figure 2).

**Table 2:** Overview of compliance, calculated as the average amount of measurements performed per day, with optimal compliance considered as 3 measurements daily.

Variable	Entire study (n=23)	First month (n=23)	Second month (n=17)	*P* value^a^
Measurements per day, mean (SD)	2.7 (0.9)	3.0 (0.8)	2.2 (0.7)	.002
Participants performing ≥3 measurements per day, n (%)	7 (30)	12 (52)	0 (0)	.001
Participants performing ≥2 measurements per day, n (%)	21 (91)	23 (100)	11 (65)	.008

^a^Obtained using a *t* test or 2-proportion z test.

On average, 2.7 (SD 0.9) measurements were performed per day. Compliance was significantly better in the 1st vs 2nd month (Table 2), and 21 participants (21/23, 91%) indicated that performing 3 measurements per day was manageable. Patients attributed the decrease in compliance mostly to feeling better and returning to work.

### Participants and Primary Care Physician’s Experience

Most participants found that using FibriCheck after discharge was reassuring and made them feel safer (17/23, 74%). Two of the participants experienced mild stress, and 1 participant experienced severe distress. Even though some participants experienced stress while performing the measurements, 20 out of 23 participants (87%) recommended it to be used in common practice. The surveyed PCPs experienced positive value by the added use of the app as a follow-up method as they felt more involved in the postoperative rehabilitation.

## Discussion

PPG technology–based smartphone apps show great promise to improve follow-up during rehabilitation after cardiac surgery. To our knowledge, this study is the first to evaluate the systematic use of a PPG-based app for remote heart rhythm monitoring in the first 1-2 months after cardiac surgery. In 23 patients with 30 days of follow-up, AF was detected in 4 (17%) patients. In 2 of these patients, the detection of AF by FibriCheck led to the diagnosis of an underlying complication. This finding suggests that late-onset or recurrent POAF after cardiac surgery may be an alarm symptom. In 1 patient, a late-onset AV block Mobitz II was detected as the irregular rhythm triggered a false positive registration of atrial flutter.

### Study Population

The population of our study consisted of a mixed cohort of surgical patients, representative for a tertiary care center. The mean age and CHA_2_DS_2_-VASc score in our population were lower compared to other short-term, invasive and noninvasive follow-up studies [14,20]. While this may indicate that our population was at a relatively low risk for AF, POAF occurred in 9 out of 23 patients (39%) during hospitalization [7]. It would be incorrect to draw conclusions from the demographic variables and compare these to the risk factors reported in the literature because of the limited number of included patients.

### Detection of Events by FibriCheck

Even with a limited sample size, our study showed an incidence of POAF after discharge of 17% (4 out of 23 participants). The value of FibriCheck is not limited to the detection of AF as it may enable early diagnosis of postoperative complications. While FibriCheck is not (yet) able to specifically detect atrioventricular conduction abnormalities, the app enabled the detection of a second-degree AV block by giving a false positive registration for atrial flutter [21]. Furthermore, this case indicates the importance of confirming the diagnosis of arrhythmias detected by PPG with a 12-lead ECG. In 1 patient in our study, POAF recurrence was most likely caused by a pericardial tamponade [22]. Likewise, we detected an episode of late-onset AF related to pneumonia. These associations suggest that AF may serve as a marker for underlying complications, supported by the established relation between AF and inflammation. In the case of pericardial tamponade or pneumonia, it is likely that a combination of hemodynamic effects and inflammatory status led to AF [23].

When comparing the first month of follow-up to the second month, several significant differences were noted. The proportion of normal or sinus rhythm measurements was significantly higher in the second month, and consequently, the numbers of warning and urgent measurements were significantly lower. This is in keeping with evidence that most recurrence arises within the first month after surgery [9,14,24].

### Strengths and Weaknesses of the App

The ability to perform measurements at any moment is one of the most attractive features of PPG-based smartphone technology, especially in a clinical culture in which remote follow-up is becoming increasingly important. It also offers a unique opportunity to study and broaden knowledge of the occurrence of postcardiac surgery complications as we observed with our patients with AV block, pericardial tamponade, and pneumonia. In our experience, FibriCheck accurately categorizes the urgency of a measurement, making it easier and less time-consuming for researchers or physicians to use the app.

Nonetheless, the implementation of PPG-based apps still faces several obstacles. First, certain arrhythmias, such as an AV block, cannot be diagnosed as such. Second, review time can cause some inconvenience, so active follow-up is warranted. When considering routine implementation of a PPG-based app into a postoperative setting, dedicated personnel is likely needed to ensure a streamlined follow-up and lower the additional workload. Basic understanding of PPG has proven useful to interpret the measurements ourselves. Finally, patient compliance, with sufficient measurements performed per day, seems essential to the success of smartphone-based PPG technology in the diagnosis and follow-up of AF. In this study, only 7 out of 23 participants (30%) performed the required 3 measurements per day, yet 21 out of 23 participants (91%) performed 2 measurements per day. As compliance decreased over time in this study, built-in reminders may ensure sufficient measurements. On the other hand, all of the arrhythmias with clinical implications diagnosed in our study were detected because patients performed a PPG registration because they were symptomatic. This highlights the importance of having a diagnostic tool readily available for patients at risk for arrhythmias, in the postoperative setting or otherwise.

### Study Limitations and Implications for Future Studies

When designing this study, it was anticipated that a large proportion of the elderly population, with a higher risk of arrhythmias, would not possess a smartphone [25] and thus be excluded from participation, thereby creating a selection bias. Indeed, the average age of included patients was rather young (mean 60.2, SD 12 years) and significantly younger than the patients excluded due to the lack of smartphone possession (mean 77, SD 8.5 years, *P***<**.001). As the fraction of adults aged 65 years or older using smartphones rose from 18% to 42% between 2013 and 2016, the implications for future studies may be diminished in the future [25]. Alternatively, future projects can actively reduce this bias by providing devices or involving caregivers.

While the small sample size of this study prevents us from offering new insights on arrhythmia occurrence after cardiac surgery, our study did find an interesting association between POAF and underlying complications. The COVID-19 outbreak (in March 2020) hindered patient inclusion and follow-up consultations. Future trials will have a larger study population and include a scaled questionnaire of patient and PCP experience to enable continued improvement of the application.

### Conclusions

This study was the first to evaluate FibriCheck in a setting after cardiac surgery. Implementation of smartphone-based PPG technology enabled the detection of AF and other (rhythm-related) complications. After discharge, even with only 23 patients included, FibriCheck detected POAF in 4 patients (17%) and a second-degree AV block in 1 patient. An association between AF detection and an underlying complication was found in 2 patients. Early detection of these complications by FibriCheck likely improved the patients’ clinical outcomes. Therefore, smartphone-based PPG technology may supplement rehabilitation after cardiac surgery by acting as a sentinel for underlying complications, rhythm-related or otherwise. With dedicated personnel and a streamlined workflow for the management of detected arrhythmias, the systemic implementation of FibriCheck into follow-up after cardiac surgery is very promising.

## Appendix

Multimedia Appendix 1 An example of a normal PPG measurement in the dashboard of the application.

## Abbreviations

AF: atrial fibrillation
AV: atrioventricular
CHA_2_DS_2_-VASc: Congestive heart failure, Hypertension, Age (>75y), Diabetes, Stroke/transient ischemic attack, Vascular disease, previous myocardial infarction, Age (65-75y) and Sex category
ECG: electrocardiogram
PCP: primary care physician
POAF: postoperative atrial fibrillation
PPG: photoplethysmography
UZ: University Hospitals
