# Supplementary material for: Remote Heart Rhythm Monitoring by Photoplethysmography-Based Smartphone Technology After Cardiac Surgery: Prospective Observational Study
Source: JMIR Mhealth Uhealth. 2021 Apr 15;9(4):e26519. doi: 10.2196/26519 (PMC8085754; doi:10.2196/26519)

Measurement Detail

Groups: UZ Leuven - Cardiochirurgie

Heartbeat: 54

Heart rhythm: Normal

Time:

Status: Reviewed

Review: Regular Rhythm

Symptoms: No Symptoms

Activity: Sitting

FibriCheck: 1.5.2 (version)

Device:

PPG signal

Download measurement | Edit Review

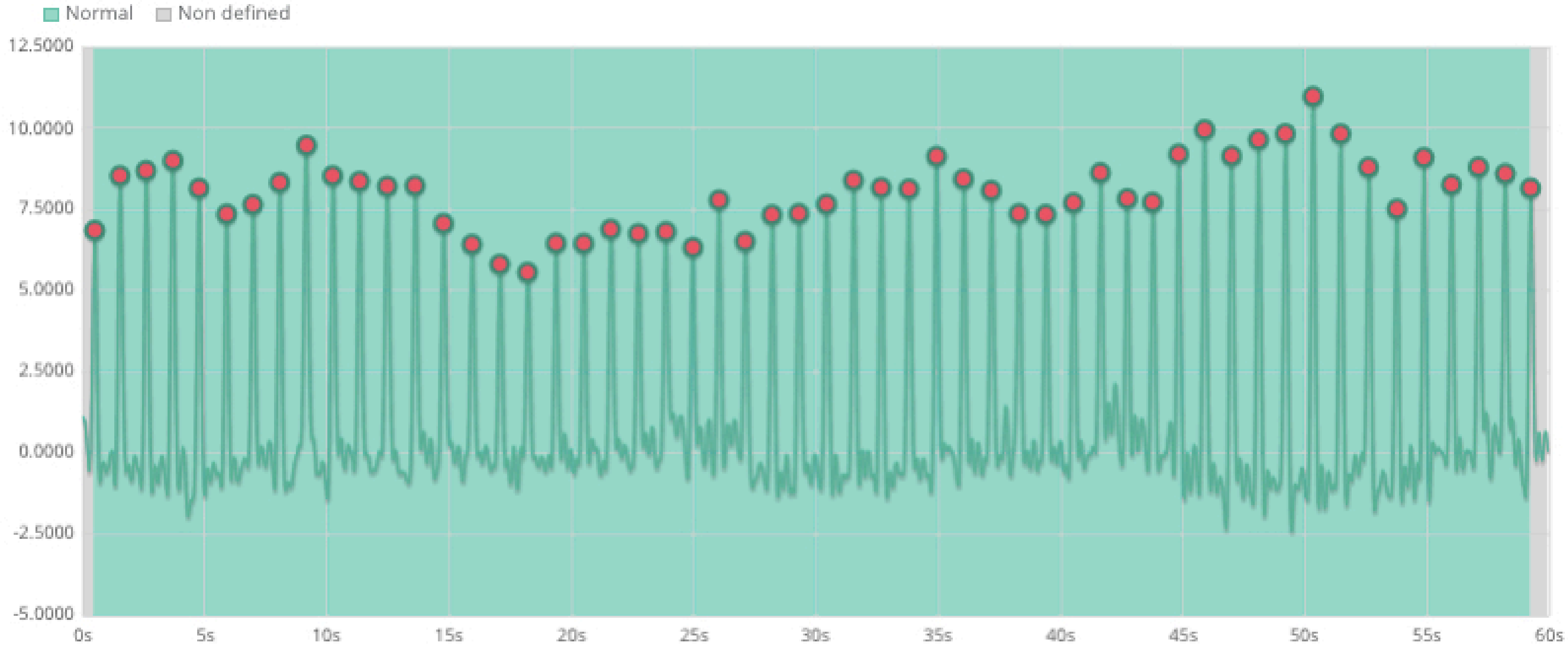

RR tachogram

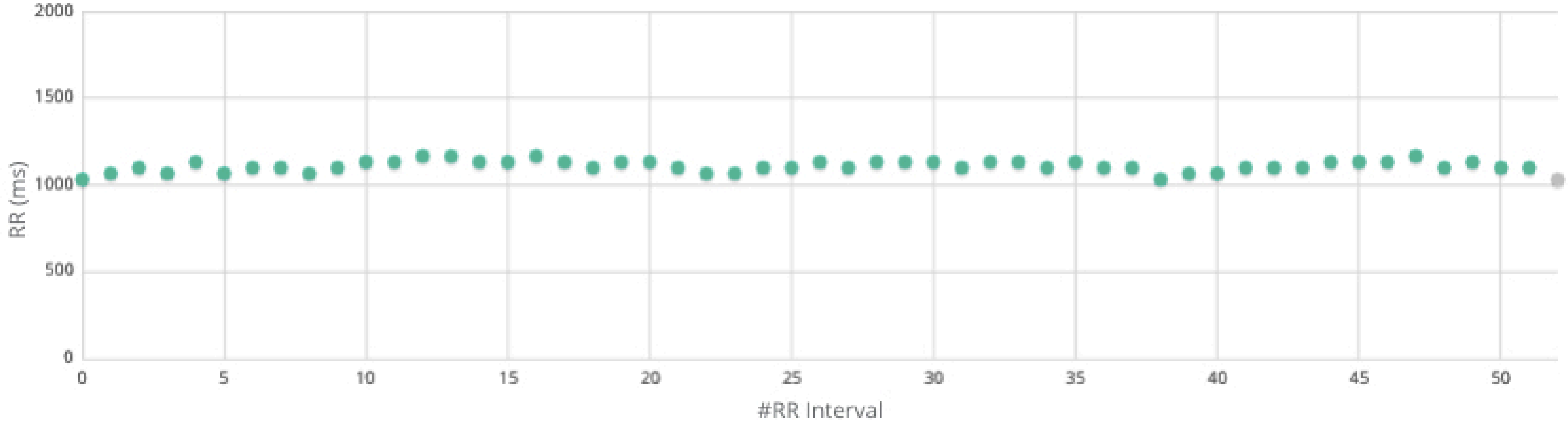

Poincaré

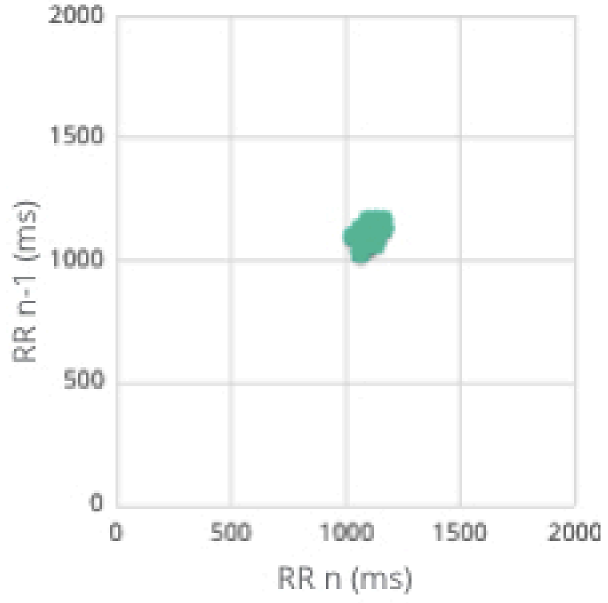

Supplement: Multimedia Appendix 1 [file mhealth_v9i4e26519_app1.pdf]
